# Hospital-based Perinatal Outcomes and Complications in Teenage Pregnancy in India

**DOI:** 10.3329/jhpn.v28i5.6158

**Published:** 2010-10

**Authors:** Prianka Mukhopadhyay, R.N. Chaudhuri, Bhaskar Paul

**Affiliations:** ^1^ Department of Community Medicine, N.R.S Medical College and Hospital, Kolkata, West Bengal, India; ^2^ Department of Maternal and Child Health, All India Institute of Hygiene and Public Health, Kolkata, West Bengal, India; ^3^ Department of Gynaecology and Obstetrics, R.G. Kar Medical College and Hospital, Kolkata, West Bengal, India

**Keywords:** Cross-sectional studies, Delivery, Obstetric, Observational studies, Pregnancy outcomes, Pregnancy in adolescence, India

## Abstract

Teenage pregnancy is a worldwide problem bearing serious social and medical implications relating to maternal and child health. A cross-sectional observational study was undertaken to compare the different sociodemographic characteristics and perinatal outcomes of teenage primigravida mothers with those of adult primigravida mothers in a tertiary-care hospital in eastern India. A sample of 350 each in cases and comparison group comprised the study subjects. Data were collected through interviews and by observations using a pretested and predesigned schedule. Results revealed that the teenage mothers had a higher proportion (27.7%) of preterm deliveries compared to 13.1% in the adult mothers and had low-birth-weight babies (38.9% vs 30.4% respectively). Stillbirth rate was also significantly higher in teenage deliveries (5.1% vs 0.9% respectively). The teenage mothers developed more adverse perinatal complications, such as preterm births, stillbirths, neonatal deaths, and delivered low-birthweight babies, when compared with those of the adult primigravida mothers. Teenage pregnancy is still a rampant and important public-health problem in India with unfavourable perinatal outcomes and needs to be tackled on a priority basis.

## INTRODUCTION

Teenage pregnancy, a social problem distributed worldwide, has serious implications on maternal and child health, especially in the context of developing countries. In India, teenage pregnancy is an important public-health problem, although the national policy of the Government of India advocates the minimum legal age of marriage for girls to be 18 years. Data of the National Family Health Survey (NFHS)-3 revealed that 16% of women, aged 15-19 years, have already started childbearing. This proportion is the highest in the state of Jharkhand (28%), followed by West Bengal (25%) and Bihar (25%), all located in eastern India. A substantial proportion of young married girls is already malnourished. Nearly 47% of adolescent women have body mass index of less than 18.5, 11.4% are stunted, and half of them have anaemia (1). While there is a growing realization of the need to promote adolescent reproductive health, work done in this field is often inadequate.

Teenage pregnancy occurs when women aged less than 20 years become pregnant. This is of serious concern because maternal age plays a significant role in adverse outcome and complications of pregnancy. Teenage pregnancies represent a high-risk group in reproductive terms because of the double burden of reproduction and growth. Complications of pregnancy and childbirth are the leading cause of mortality among girls aged 15-19 years in developing countries (2).

The combination of poor nutrition and early child bearing expose young women to serious health-risks during pregnancy and childbirth, including damage to the reproductive tract, pregnancy- related complications, such as anaemia, pregnancy-induced hypertension, preterm labour, cephalopelvic disproportion, maternal mortality, perinatal and neonatal mortality, and low birthweight (3,4). Industrialized and developing countries have distinctly different incidences of teenage pregnancy. In developed regions, teenage mothers tend to be unmarried, and adolescent pregnancy is seen as a social issue whereas, in developing countries, such pregnancies mostly occur in married teenagers, and their pregnancy is most often welcomed by family and society. However, in these societies, early pregnancy may combine with malnutrition and poor healthcare to cause medical problems.

Studies on complications in teenage pregnancy have yielded conflicting results, and opinions of different authors vary in this regard. Some have opined that age by itself is not a risk factor, and poor outcomes are associated more with socioeconomic factors rather than with biological factors (5). Other researchers have failed to find any evidence for major impairments of pregnancy outcome among teenage mothers with provision of high-quality maternal care with complete coverage (6).

With greater understanding of the antecedents of teenage pregnancy, especially in the context of developing countries like India, it may be possible to develop more effective interventions to tackle this widespread problem. Most studies in India have used record-based data. There is a lack of recent data on the perinatal outcomes of teenage pregnancy in eastern India under the changing scenario of socioeconomic development and availability of better healthcare facilities.

The objective of the present study was to compare the sociodemographic characteristics and perinatal outcomes of teenage mothers with those of adult primigravida mothers aged 20-29 years attending a tertiary-care hospital for their deliveries.

## MATERIALS AND METHODS

### Study setting and design

The study was conducted at the R.G. Kar Medical College and Hospital in Kolkata during June 2006–May 2007. This is one of the busiest tertiary-care hospitals in eastern India, with over 10,000 deliveries annually. The study undertaken was of a cross-sectional, observational type with two groups—cases and comparison—respectively.

Teenage pregnancy was defined as pregnancy occurring during the maternal ages of 13-19 completed years at delivery. Primigravida teenage mothers aged 13-19 years were regarded as the cases while primigravida adult mothers aged 20-29 years formed the comparison group. Primigravida women were selected to eliminate the influence of parity. Age between 20 and 29 years was considered since this age-group is generally regarded as safe for childbirth. Ages above 30 years constituting elderly primigravida, multiple gestation, women with major illnesses during pre-pregnant state, and any perinatal complication occurring after 48 hours of delivery were excluded from the study.

Low-birthweight baby has been found to be an important outcome of teenage pregnancy. According to a hospital-based case comparison study, 72.6% of low-birthweight babies were born to teenage mothers compared to 59.2% of low-birthweight babies in the comparison group, comprising adult primigravida mothers aged 20-29 years (7).

The Epi Info software (version 3.3.2) was used for calculating the sample-size. Using the two proportions and at 95% confidence interval, power of 95% and 1:1 comparison, the number of subjects in each group was calculated to be 337. In the present study, a sample of 350 each in cases and comparison group, with a total of 700 mothers comprised the study subjects.

The principal investigator visited the labour-wards, labour-room, and operation theatre on three days in a week as chosen randomly and those days were fixed for data collection and were applicable throughout the period of data collection. Since cases admitted for delivery were not evenly distributed over the days, on the working days of study visit, an average of three consecutive cases/day of deliveries by teenage mothers were considered. For each case, next consecutive singleton delivery in the age-group of 20-29 years was selected for comparison.

A pilot study was done on 20 women before undertaking the actual study to find out the validity of the schedule used for interviewing the study subjects.

### Collection and analysis of data

Data were collected through interviews and by observations using the pretested and predesigned schedule. The first contact with the study participants for data collection began immediately after delivery of the baby. Mode of delivery and perinatal outcomes were observed, and Apgar score was calculated at the first minute and the fifth minute after birth. The study participants were interviewed using the schedule as soon as their condition permitted, and in the cases where they had not recovered, their closest kin was interviewed. Data were verified, and missing information was collected from the antenatal records, if available. Data were compiled and analyzed using the MS Excel and Epi Info software (version 2002). Statistical tests used were chi-square and Student's *t*-test, and the p value of <0.05 was considered significant.

### Ethics

Informed verbal consent was obtained from all the study subjects before they went into active labour.

The procedures followed were in accordance with the ethical standards of the Committee on Human Experimentation of the R.G. Kar Medical College and Hospital.

## RESULTS

### Sociodemographic characteristics

The maximum number of teenage mothers (age 13-19 years) belonged to the age-group of 18-19 years (approximately 89%). There was no teenage mother aged less than 15 years. Their mean (±SD) age was 18.3±0.8 years. The maximum number of adult mothers (age 20-29 years) belonged to the age-group of 20-21 years (approximately 41%), and only 7% belonged to the age-group of 28-29 years. Their mean (±SD) age was 22.8±2.6 years. The different sociodemographic characteristics are summarized in Table 1. Illiteracy and joint-family structure were significantly associated with teenage pregnancy. A higher proportion (72.9%) of the adult mothers (n=350) received a minimum of three antenatal check-ups compared to the teenage mothers (68.6%). The more number (78.86%) of the adult mothers completed their full course of two doses of tetanus toxoid than the teenage mothers (74.29%). The adult mothers had a higher proportion (49.1%) consuming more than 100 iron folic acid tablets compared to the teenage mothers (40%), and the association between the age-group of mothers and the consumption of 100 iron folic acid tablets was significant (p<0.05). The ever-use of any contraceptives was significantly higher among the adult mothers (18.8%) compared to only 1.7 % among the teenage mothers.

**Table 1.** Comparison of teenage and adult primigravida mothers according to different sociode-mographiccharacteristics

Sociodemographic characteristics	Teenage mothers (n=350)		Adult primigravida mothers (n=350)		p value
	No.	%	No.	%	
Literate	186	53.1	210	60.0	<0.05
Joint family	273	78.0	246	70.3	<0.05
Marital status	350	100.0	348	99.4	>0.05
Addiction to tobacco	10	2.9	13	3.7	>0.05
Antenatal care					
Early registration	198	56.6	252	72.0	<0.05
≥3 antenatal visits	240	68.6	255	72.9	>0.05
≥100 iron folic acid tablets consumed	140	40.0	172	49.1	<0.05
2 doses of tetanus	260	74.3	276	78.8	>0.05
Height <140 cm	61	17.4	58	16.6	>0.05
Ever-use of contraceptives	6	1.7	66	18.8	<0.05

### Outcomes of pregnancy

The outcomes of deliveries are presented in Table 2. The teenage mothers had a higher proportion (65.7%) of normal vaginal delivery compared to the older mothers (61.4%). This could be due to a higher proportion of smaller babies in that age-group. About 34% of the teenage mothers had instrumental delivery (forceps and caesarean) compared to 39% of the adult mothers. However, the association between the age of mothers and the mode of delivery was not significant (p>0.05). The adult mothers had a higher proportion (7.7%) of post-term pregnancies compared to the teenage mothers (2%). The teenage mothers had a higher proportion (27.7%) of preterm deliveries compared to the adult mothers (13.1%). The association between the age at conception and the period of gestation during delivery was significant. Stillbirth rate was significantly higher in teenage deliveries (5.1% vs 0.9% in the comparison group). Teenage pregnancy was significantly associated with low birthweight (<2.5 kg). The mean birthweight was 2.59 kg in the teenage-group and 2.72 kg in the control group, and the difference was highly significant.

**Table 2.** Comparison of teenage and adult primigravida mothers according to outcomes of delivery

Outcome of delivery	Teenage mothers (n=350)		Adult primigravida mothers (n=350)		p value
	No.	%	No.	%	
Presentation of baby					
Vertex	344	98.3	339	96.9	>0.05
Breech	6	1.7	11	3.1	>0.05
Mode of delivery					
Vaginal	230	65.7	215	61.4	>0.05
LSCS	99	28.3	129	36.9	>0.05
Forceps	21	6.0	6	1.7	>0.05
Period of gestation at delivery					
Preterm	97	27.7	46	13.1	<0.05
Post-term	7	2.0	27	7.7	<0.05
Stillbirths	18	5.1	3	0.9	<0.05
Low birthweight	137	38.9	108	30.4	<0.05

LSCS=Lower segment caesarian section

Table 3 compares the neonatal complications among the teenage and adult primigravida mothers. Although birth-asphyxia was the most common neonatal complication in both the groups, it was significantly higher in the teenage-group compared to their older counterparts. Deaths of newborns within 48 hours were also higher in the case of the teenage mothers.

**Table 3.** Comparison of teenage and adult primigravida mothers according to complications of their newborns

Complications of newborns	Teenage mothers (n=350)		Adult primigravida mothers (n=350)		p value
	No.	%	No.	%	
Birth-asphyxia	58	16.6	31	8.9	<0.05
Sepsis	14	4.0	10	2.9	>0.05
Neonatal jaundice	16	4.6	12	3.4	>0.05
Congenital anomalies	3	0.9	2	0.6	>0.05
Deaths within <48 hours	18	5.1	6	1.7	<0.05

## DISCUSSION

Although the legal age at marriage is 18 years for females and 21 years for males in India, early marriage is common. By the age of 15 years, 26% of females are married, and by the age of 18 years, this figure rises to 54%. Most reproduction in India occurs within marriage; so, the low age at marriage automatically links to early onset of sexual activity and thereby fertility (8). Pregnancies occurring outside wedlock have the risk of terminating in unsafe abortions by quacks and often do not reach the tertiary hospital. In this study, all the adult mothers were married, and only two teenage mothers were unmarried.

Teenage pregnancies put mothers at high risk to many health-related complications and their newborns to poor birth outcomes. Adverse outcomes of teenage pregnancy arise not only from physical and medical causes but are also associated with individual, familial and sociocultural factors besides lack of access to healthcare, contraception, and other resources which is the prevailing situation in most developing countries. This study aimed at finding the distribution of different sociodemographic characteristics, such as education, type of family, etc., between teenage mothers and their older counterparts. A significantly higher proportion (78%) of the teenage mothers belonged to joint families compared to the older mothers (70%). This could be explained by the fact that the teenage women in a joint family remain under family pressure from their in-laws and cannot decide to bear children according to their own desire. Low levels of literacy adversely affect reproductive and sexual health awareness and, thus, quality of life. An early start of childbearing greatly reduces the educational and employment opportunities of women and is associated with higher levels of fertility. In this study, the literacy rate was higher among the adult mothers compared to the teenage mothers, and the association between the age at conception and the literacy status was significant. Contraceptive-use was significantly higher among the adult mothers (18.9%) than among the teenage mothers (1.7%). Contraceptive-use was much lower among the teenage population possibly because of their lower levels of education and family pressure for childbearing. Early registration by 16 weeks was lower among the teenage-group. Antenatal care received was, overall, similar in both the groups. However, early registration of pregnancy and consumption of the recommended number of prophylactic iron folic acid tablets were significantly lower among the teenage mothers. This indicates that the teenage mothers were less careful about their pregnancy probably because of the lack of awareness and maturity. Other authors have reported early registration of pregnancy ranging from 40% to 90% in teenagers; however, the frequency of antenatal check-ups by them was consistently lower (7,9,10–13).

Regarding the outcomes of pregnancy, the majority of the deliveries had vertex presentation, and the comparison group had a higher proportion of breech delivery (3.1%) compared to the teenage mothers (1.7%) but the association was not significant. No transverse lie was found in any age-group. About two-thirds of the babies were born by normal vaginal delivery. The teenage mothers had a higher proportion of normal delivery compared to the older mothers. This could be due to a higher proportion of smaller babies in that age-group. About 34% of the teenage mothers in this study had instrumental delivery compared to 39% of the adult mothers. However, the association between the age of mothers and the mode of delivery was not significant. The most common overall indication for caesarean section was foetal distress (about 60%), followed by cephalopelvic disproportion (22.8%). Indication for caesarean section in foetal distress and pre-eclampsia was more commonly found among the teenage mothers than among the adult mothers. Opinion on modes of delivery by operative intervention in teenage pregnancy differed widely. Some authors have reported a higher rate of instrumental deliveries in the case of teenage pregnancies (14–16). The possible explanation could be underdevelopment of pelvis in younger mothers and occurrence of cephalopelvic disproportion more frequently in teenage mothers; consequently, the number of instrumental deliveries and caesarean sections was also higher. Other authors have reported lower rates, and some contradicted this view (17–19). The higher frequency of occurrence of low-birthweight babies in the teenage-group than controls is the most common reason in such cases. The teenage mothers had a significantly higher number of preterm deliveries compared to the adult mothers while the reverse was noted in post-term deliveries. Such a high incidence of preterm labour leads to higher risks for neonates. Many authors from developed countries have reported an association between teenage pregnancy and preterm delivery (20–24). Some authors from India have reported similar findings (7,9,23,25–28).

Low birthweight is a key predictor of malnutrition and an important determinant of child mortality (29). One of the most detrimental outcomes of low birthweight is growth retardation, and if the newborn happens to be a girl, it perpetuates a vicious cycle of female malnutrition throughout adolescence and adulthood. This process gives rise to a condition of intergenerational transmission of physical (small mothers have small babies), social and economic disadvantages into the next generation (30). The present study found that the number of low-birthweight babies was more in the case of teenage mothers (38.9%) compared to the adult mothers (30.4%). Babies born to teenage mothers are likely to be premature, and hence, the incidence of low birthweight is higher in them. This observation corroborates the findings of several other authors (7,12,23,31–33).

The proportion of stillbirths was also higher (5.1%) among the teenage pregnancies. Of the neonatal complications, neonatal deaths and birth-asphyxia were significantly higher in the teenage-group compared to the adult primigravida women possibly due to the more number of premature births. Occurrences of other complications in the two groups were not different. However, some bias might have been introduced, as the complications delayed beyond 48 hours could not be observed since there was no scope of any follow-up.

### Limitations

The primary limitation of the study was that, since it was conducted in a tertiary-care hospital set-up, chances of high-risk cases may be more, and it may not truly reflect the prevailing situation in a community setting. Family income, which can be an important determinant in pregnancy outcomes, could not be included under sociodemographic characteristics, as income could not be verified. Another limitation of the study was that the findings of adverse perinatal outcomes of teenage pregnancy could have been confounded by the unequal distribution of different sociodemographic characteristics in the two groups. Further studies are needed to quantify the adverse outcomes after adjusting for the different confounding factors.

### Conclusions

The present study was an attempt to throw light on the different sociodemographic characteristics relating to teenage pregnancy and their outcomes and complications compared to older (20-29 years) primigravida mothers. In general, it was found that the teenage mothers were from a socioeconomically-disadvantaged background with lower levels of education and used lesser antenatal healthcare services. They developed more perinatal complications, such as preterm births, stillbirths, and neonatal deaths, and delivered babies with low-birthweight compared to the older mothers. The adverse outcomes of teenage pregnancy could be attributed not only to lower maternal age but also to their relatively-disadvantaged socioeconomic background. To address this multifaceted problem, we should aim to reduce the incidence of teenage pregnancy, not only to minimize the adverse outcomes on young mothers but also to limit the family-size. Efforts need to be directed towards strict enforcement of laws prohibiting teenage marriage in India. Access to quality health services that are gender-sensitive and adolescent-friendly should be ensured. For pregnant adolescents attending the antenatal clinic, extra care should be taken to ensure that the minimum number of regular antenatal visits is made. Appropriate and adequate counselling on different antenatal services are to be offered to them. Tetanus toxoid immunization and consumption of the recommended dose of iron and folic acid should be checked, and intake of an additional meal should be advised at every visit. In addition, they should be advised to take more rest and have adequate sleep to avoid premature births. Smoking, alcohol-use, and other addictions should be strongly discouraged. Early detection of complications, such as anaemia, pre-eclampsia, and intrauterine growth restriction and their management, and good intranatal and postnatal care are essential. Contraceptive practices need to be promoted among married adolescents so that future pregnancy could be delayed till they reach maturity. Steps should be taken to educate adolescent mothers about the health hazards of too early and repeated successive pregnancies. Teenage pregnancy needs to be tackled as a priority to ease the burden of socioeconomic and health problems.
